# Arthroscopy assisted single bone tunnel two stranded en Masse repair for peripheral triangular fibrocartilage complex tears

**DOI:** 10.1038/s41598-025-28681-4

**Published:** 2025-12-29

**Authors:** Won-Taek Oh, Hee-Soo Kim, Hyun-Kyo Kim, Jae-Yong Cho, Il-Hyun Koh, Yun-Rak Choi

**Affiliations:** Department of Orthopedic Surgery, Yonsei University College of Medicine, 50-1, Yonsei-ro, Seodaemun-gu, Seoul, 03722 Korea

**Keywords:** Triangular fibrocartilage complex, Peripheral tear, Arthroscopic repair, En masse repair, Outcomes research, Ligaments

## Abstract

This study aimed (1) to provide a stepwise, reproducible description of a single-bone-tunnel, two-stranded, anchorless ligament-specific (‘en Masse’) repair for traumatic peripheral TFCC tears, and (2) to evaluate one-year clinical outcomes and exploratory prognostic factors in a retrospective consecutive case series. Forty-eight patients were retrospectively included. Postoperatively, ballottement testing revealed no detectable distal radioulnar joint (DRUJ) laxity (*p* < 0.001). The mean visual analog scale (VAS) pain score (7.2 to 1.9), grip strength (66.1% to 81.3%), Mayo wrist score (MWS, 64.3 to 81.9), and Disabilities of the Arm, Shoulder, and Hand (DASH) score (37.1 to 12.7) were improved postoperatively (*p* < 0.001). There were no surgery-related complications. On multivariable analysis, longer symptom duration (*p* = 0.031) and positive ulnar variance (*p* = 0.045) were associated with lower MWS; model fit was modest (*R*^2^=0.224). Arthroscopic-assisted en Masse repair for peripheral TFCC tears was associated with improved clinical outcomes at one year or more after surgery, and postoperative ballottement testing revealed no detectable DRUJ laxity. Within this cohort, one-year follow-up precluded assessment of long-term durability; also, applicability to degenerative/chronic peripheral TFCC tears remains undetermined.

**Supplementary Information:**

The online version contains supplementary material available at 10.1038/s41598-025-28681-4.

## Introduction

Due to unconstrained articulation between the sigmoid notch of the radius and the ulnar head, the triangular fibrocartilage complex (TFCC) is the most responsible for the distal radioulnar joint (DRUJ) stability^1,2^. An intrinsic TFCC comprises a triangular fibrocartilage (TFC) and palmar and dorsal radioulnar ligaments (RUL), and the RUL composes peripheral TFCC, which is separated into a deep limb attached to the ulnar fovea and a superficial limb attached to the ulnar styloid^3–5^. The deep limb of TFCC, due to an obtuse insert angle, plays a crucial role in stabilizing DRUJ during distal radioulnar rotation, and surgical repair is required when it is completely ruptured^1,6,7^.

Arthroscopic-assisted TFCC repair is gradually occupying a large portion of the treatment for TFCC peripheral tears, and various repair techniques have been introduced^2,3,8,9^. It was first established by Nakamura et al. based on two bone tunnels to the ulnar fovea and one strand to fix TFCC^2^. After that, clinical experts have endeavored to make repair methods simpler and more specific. Achieving an optimal foveal tunnel position is difficult to reproduce consistently^10^, and creating two tunnels with distinct, precise trajectories is even more technically demanding. Bone-tunnel–related complications—including iatrogenic ulnar styloid fracture and postoperative ulnar-sided wrist pain attributable to tunnel remodeling—have also been reported^11,12^. In this context, several groups have proposed a single-tunnel strategy, often using a wide tunnel (over 4 mm) that permits multiple suture trajectories^8,9^. Within such tunnels, sutures can be passed at two or more angles to capture the TFCC; however, an anchor is typically required for final tensioning. Across comparative studies, anchor-based repairs have not shown higher overall complication rates than suture-based transosseous repairs^13,14^; nevertheless, anchor migration^15^ and anchor-related foreign-body reaction and osteolysis have been reported^16,17^. Meanwhile, a ligament-specific repair was also introduced, using two separate strands to secure both palmar and dorsal RUL, although it required two bone-tunnels with a more precise angular configuration^3^.

Building on the movement toward simpler and ligament-specific constructs, the single-bone-tunnel, two-strand (or more), anchorless arthroscopic repair has gained increasing clinical interest^18^. Although operative details may differ among surgeons, this approach is comparatively straightforward (single-bone-tunnel), ligament-specific (two or more strands addressing the palmar and dorsal RUL), and anchorless (thus avoiding anchor-related complications). In this manuscript, we provide a stepwise, reproducible description of a single-tunnel, two-strand, anchorless repair; two strands—each capturing both the superficial and deep components of its respective RUL (dorsal or palmar)—achieve an en masse construct, and we refer to this technique as an “en Masse” repair. We also report one-year clinical outcomes and explore prognostic factors in a retrospective consecutive series.

## Methods

### Study design and objectives

We conducted a retrospective consecutive case series to describe a single-bone-tunnel, two-strand, anchorless ligament-specific TFCC repair technique and to evaluate one-year clinical outcomes and exploratory prognostic factors.

### Participants and eligibility criteria

From October 2015 to May 2021, we screened 60 consecutive patients who underwent arthroscopic-assisted en Masse repair for TFCC peripheral tears. We included (1) traumatic peripheral TFCC tears involving the deep limb of TFCC (Atzei class 2 or 3)^19^ and (2) follow-up at least 12 months after the operation. After applying the eligibility criteria, 12 patients were excluded: three with isolated superficial limb tear (Atzei class 1), three with degenerative lesions either in the ulnocarpal joint or DRUJ, two revision TFCC repairs, and four with less than 12 months of follow-up. A single hand surgeon conducted all surgeries during the study period. All patients’ medical records and radiographs were retrospectively reviewed. Our institutional review board approved this study, waiving informed consent. (Institutional Review Board, Severance Hospital, Yonsei University Health System, Approval No. 4-2023-1443). All methods were performed in accordance with the relevant guidelines and regulations.

### Surgical technique

**Fig. 1:**
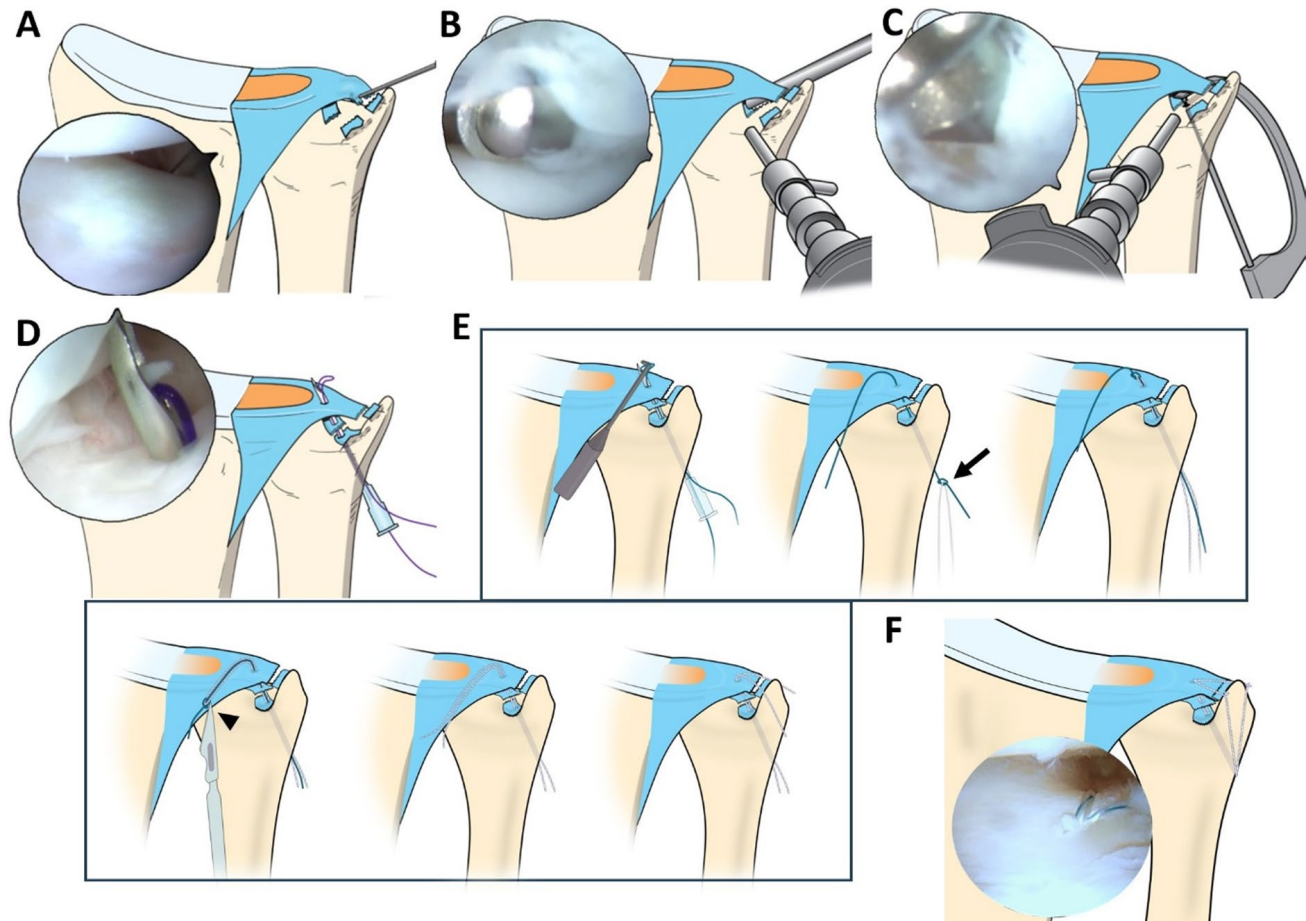
Surgical procedures of arthroscopic en Masse repair for Triangular fibrocartilage complex (TFCC) peripheral tear. (**A**) The foveal detachment of TFCC was identified using the hook test via a 3–4 portal as a viewing portal and a 6-R as a working portal. (**B**) The ulnar fovea was debrided to enhance the healing potential through a distal radioulnar joint (DRUJ) portal as a viewing portal and a direct foveal portal as a working portal. (**C**) A bone tunnel was created using a 0.9-mm K-wire, then a 1.1-mm K-wire with the assistance of the TFCC guide. (**D**) A 20-gauge needle was inserted through the bone tunnel and pierced the TFCC, and a 2 − 0 PDS suture was passed through the needle. (**E**) The PDS was replaced with the nonabsorbable 2 − 0 FiberWire while make the FiberWire two-strands by tying the PDS in the middle of the FiberWire (arrow) and cut one-half after passing out of the 6-R portal (arrowhead). (**F**) Each suture were tied, with the wrist placed in a neutral rotation position. The FiberWire suture in the dorsal 6-U portal fastened the dorsal superficial and deep limbs of TFCC together, and the volar FiberWire fastened the palmar segments. Figure created with Inkscape v1.4.2 (https://inkscape.org).

Under general anesthesia and with the patient supine, the patient’s arm was prepped and draped on a hand table. An Esmarch bandage and tourniquet were used to exsanguinate the arm. The patient’s arm was then suspended in an Arc Wrist Tower (Acumed, Hillsboro, OR, USA) with 5–8 kg of traction after placing the index, middle, and ring fingers in finger traps. The standard 3–4 and 6-R arthroscopy portals were created sequentially, and a 1.9-mm video arthroscope was introduced through the 3–4 portal for visualization. The foveal detachment was identified using the trampoline and hook tests (Fig. 1A)^20^. When the results of these tests were suspicious of a foveal tear, the distal DRUJ and the direct foveal portal were created, as previously described.

An arthroscope was introduced through the DRUJ portal, and the fovea was examined. A 2.0-mm motorized shaver was inserted through the direct foveal portal to debride the fovea and enhance the healing of the TFCC after the repair (Fig. 1B). A 2-cm longitudinal incision was made from the ulnar styloid tip in a distal to proximal direction. The extensor retinaculum was released, and the ulnar side of the distal ulna was exposed while protecting the dorsal sensory branch of the ulnar nerve. A specifically designed TFCC guide (C-Ring Aiming Guide: Arthrex Inc., Naples, FL, USA) was inserted through the direct foveal portal. The guide’s pointed tip was seated directly on the ulnar fovea (footprint) to define the Kirschner wire (K-wire) exit point and maximize the accuracy of foveal tunnel placement, rather than positioning the guide above the TFCC. While keeping the tip of the guide on the fovea, we placed the proximal part of the guide on the ulnar cortex at a location 1.5 cm proximal to the ulnar styloid tip. One 0.9-mm K-wire was inserted through the shaft, piercing the fovea (Fig. 1C). The guide and K-wire were then removed. The bone tunnel was widened using a 1.1-mm K-wire. A 20-gauge needle was inserted through the bone tunnel into the fovea, and the torn TFCC was pierced while the cut side faced the distal and ulnar to allow the needle to penetrate the TFCC vertically. The needle tip was 20 degrees bent to center the tip for precise penetration. Arthroscopy was then inserted via the 3–4 portal to find the needle tip, which came through the TFCC into the ulnocarpal joint. Next, a 2–0 polydioxanone (PDS) suture was passed through the needle and out of the ulnocarpal joint through the 6-R portal (Fig. 1D). Only the needle was removed, leaving the PDS suture in place. The PDS suture was replaced with a 2 − 0 FiberWire suture using a shuttle relay technique. To make the FiberWire two strands, the prepositioned PDS was tied in the middle of the FiberWire suture, and the FiberWire suture was cut one-half after passing out of the 6-R portal (Fig. 1E). Two additional 6-U portals were created within the previous ulnar skin incision, volar and dorsal to the ulnar styloid, and each FiberWire suture was separately retrieved through different 6-U portals. Then, FiberWire sutures were tied, with the wrist placed in a neutral rotation position. The FiberWire suture in the dorsal 6-U portal fastened the dorsal superficial and deep limbs of TFCC together, and the volar FiberWire fastened the palmar segments (Fig. 1F). Finally, we confirmed the stable repair of the torn TFCC using the trampoline and hook tests. The entire surgical procedures are extensively described in Supplementary Video 1. Patients were encouraged to initiate immediate digital exercises to reduce swelling. Immobilization with a short arm brace was continued for an additional 2 weeks. Six weeks after surgery, passive-assisted wrist movement exercises were begun. Strengthening exercises of the forearm and wrist were started after 3 months, and heavy physical activities were allowed 6 months after surgery.

### Clinical and radiological assessments

The instability of the DRUJ was assessed by an orthopedic surgeon (Y.C) at an outpatient clinic preoperative and in the postoperative period using the ballottement test as described in earlier articles^21,22^. Briefly, the patient’s forearm was upright in the neutral position with the patient’s elbow placed on the table. An examiner held the radius and carpus with one hand and moved the ulnar head dorsal and palmar direction with the contralateral hand. The instability was graded 0–3 relative to the contralateral wrist: Grade 0, stable joint without laxity; Grade 1, suspicious laxity compared to contralateral; Grade 2, remarkable laxity with a firm endpoint; Grade 3, remarkable laxity without a firm endpoint^23^. Because all assessments were performed by a single surgeon, inter-observer reliability was not assessed.

One observer (H.C) evaluated the following clinical outcomes preoperatively and during the follow-up: visual analog scale (VAS) pain score, grip strength, Mayo wrist score (MWS), Disabilities of Arm, Shoulder, and Hand (DASH) score, and active range of motion of the wrist. The VAS pain score was stratified from 0 (no pain) to 10 (the worst pain), depending on the severity of pain experienced by patients. Grip strength was measured using a Jamar hydraulic dynamometer (Asimov Engineering, Los Angeles, CA)^24^. The DASH score is based on self-reported answers to a questionnaire designed by Davis et al., which contains 30 items: 21 questions that assess difficulties with specific tasks, 5 questions that evaluate symptoms, and 4 questions that assess social function, work function, sleep, and confidence^24^. The DASH score ranges between 0 and 100, with higher scores representing greater upper extremity disability. Active flexion-extension, supination-pronation, and radial-ulnar deviation arcs of the wrist were measured using a handheld goniometer. Postoperative complications and the need for secondary surgery were also reviewed.

The prognostic factors of poor postoperative clinical outcomes were analyzed with MWS and DASH score as dependent factors. Independent factors included the patient’s age, sex, symptom duration, ulnar positive variance, presence of the ulnar styloid fracture, and a combined central or superficial limb tear of TFCC. The ulnar variance was measured in a preoperative pronated grip view of the wrist using the method of perpendiculars by Palmer^25^. The previous ulnar styloid fracture was confirmed in preoperative plain radiographs of the wrist. The combined central or superficial limb tear of TFCC was investigated via captured arthroscope images (or arthroscope video files) during the intraoperative arthroscopic examination. Two orthopedic surgeons (W.O, H.K) evaluated all radiographic and intraoperative factors.

All data were obtained from medical records, and no information was missing. No patient was recalled to our institution specifically for this study. Our institutional review board approved this study, waiving informed consent.

### Statistical methods

Data are presented as the mean ± standard deviation (SD) unless otherwise indicated. All statistical computations relied on standard software (R freeware v4.2.0; R Foundation for Statistical Computing, www.r-project.org). The Cochran-Armitage trend test was used to analyze the postoperative improvement of DRUJ stability. The paired *t*-test or Wilcoxon signed-rank test was used to compare pre- and postoperative VAS pain score, grip strength, MWS, DASH score, flexion-extension, supination-pronation, and radial-ulnar deviation arcs. Multiple regression analyses were performed with MWS and DASH scores to evaluate prognostic factors affecting poor clinical outcomes. The level of significance was set at *p* < 0.05. This retrospective consecutive case series did not undergo an a priori sample size calculation; all eligible patients were included (*n* = 48). For paired pre-post analyses, we report mean changes with 95% confidence intervals (CI) and within-subject standardized effect sizes (Cohen’s *dz* = mean change / SD of change). A sensitivity statement indicates that with *n* = 48, two-sided *α* = 0.05, and 80% power, the minimal detectable within-subject standardized effect is *dz* ≈ 0.40 (moderate). For multivariable linear regression, analyses were exploratory. We summarize the model’s overall effect using Cohen’s *f* ^2^ = *R*^2^ / (1 − *R*^2^) and provide a sensitivity statement for the joint test of k predictors. With *n* = 48, *k* = 7 predictors, and *α* = 0.05 (df_1_ = 7, df_2_ = 40), power to detect a medium overall effect (*f* ^2^ = 0.15) is approximately 0.32, and power to detect the observed overall effect (*R*^2^ = 0.224; *f* ^2^ ≈ 0.289) is approximately 0.60.

## Results

A total of 48 patients were included from an initial 60 screened. Exclusion comprised Atzei class 1 (*n* = 3), degeneration in the ulnocarpal joint (*n* = 3), revisional TFCC (*n* = 2), and inappropriate follow-up (*n* = 4). The mean age was 31.1 ± 11.6 (19–59) years, and the average duration of follow-up was 23.9 ± 12.7 months. Thirty-seven were male, and 14 were Atzei class 2 (29.2%). Clinical outcomes before and after surgery were summarized in Table 1. MWS improved from 64.3 ± 15.2 preoperatively to 81.9 ± 10.0 postoperatively (mean change = 17.6; 95% confidence intervals (CI) = 13.5 to 21.7; *dz* = 1.25; *p* < 0.001), and the DASH score decreased from 37.1 ± 14.3 to 12.7 ± 7.7. The DRUJ stability assessed by the ballottement test revealed no detectable DRUJ laxity after the operation (mean change = -24.5; 95% CI = -28.9 to -20.1; *dz* = 1.62; *p* < 0.001). The range of motion, including the wrist flexion–extension arc, showed no significant change (Table 1). There were no surgery-related complications. One patient underwent a secondary ulnar shortening osteotomy for persistent ulnar-sided wrist pain after the primary operation. In multivariable linear regression, the model explained 22.4% of the variance in postoperative MWS (*R*^2^=0.224). Longer symptom duration (*p* = 0.031) and positive ulnar variance (*p* = 0.045) were associated with lower MWS (Table 2).

**Table 1:** Preoperative and postoperative clinical outcomes.

Variables	Preoperative	Postoperative	P**
DRUJ instability (n)*			< 0.001^†^
Grade 0	0	44	
Grade 1	15	4	
Grade 2	30	0	
Grade 3	3	0	
VAS pain	7.2 ± 1.7	1.9 ± 1.7	< 0.001^†^
Grip strength (%)	66.1 ± 17.7	81.3 ± 15.5	< 0.001^†^
MWS	64.3 ± 15.2	81.9 ± 10.0	< 0.001^†^
DASH score	37.1 ± 14.3	12.7 ± 7.7	< 0.001^†^
Flexion-extension arc (°)	141.5 ± 12.2	142.7 ± 12.6	0.242
Supination-pronation arc (°)	166.3 ± 8.5	165.9 ± 12.1	0.999
Radial-ulnar deviation arc (°)	50.6 ± 6.3	51.2 ± 4.9	0.559

Continuous values are mean ± standard deviation. Percentage values are percentages of the contralateral side.

*MWS* Mayo wrist score, *VAS* visual analog scale, *DASH* disabilities of arm, shoulder, and hand.

*DRUJ instability was measured by the ballottement test.

** P values are calculated using the Cochran-Armitage trend test for categorical variables and the paired t-test or the Wilcoxon signed-rank test for continuous variables.

^†^*P* < 0.05.

**Table 2:** Factors associated with postoperative clinical outcomes (multivariable linear regression).

Variables	MWS				DASH score			
	B	SE	t	*P* >|t|	B	SE	t	*P* >|t|
Age (yr)	0.10	0.15	0.68	0.504	-0.17	0.12	-1.34	0.187
Sex	1.08	4.11	0.26	0.794	4.57	3.39	1.35	0.186
Duration (mo)	-0.16	0.07	-2.23	0.031^†^	0.03	0.06	0.58	0.568
Positive UV	6.11	2.95	2.07	0.045^†^	-1.10	2.43	-0.45	0.653
DRUJ instability*	4.79	2.82	1.70	0.097	0.61	2.33	0.26	0.795
TFCC, intraoperative								
Central tear	0.25	0.08	0.94	0.938	1.21	2.66	0.45	0.653
Superficial tear	0.65	0.20	0.84	0.841	-3.36	2.65	-1.27	0.213
*N*	48				48			
*R* ^*2*^	0.224				0.100			
*Adjusted-R* ^*2*^	0.089				0.058			
*P*	0.149				0.727			

*MWS* Mayo wrist score, *DASH* disabilities of arm, shoulder, and hand, *UV* ulnar variance, *DRUJ* distal radioulnar joint, *TFCC* triangular fibrocartilage complex, *B* unstandardized coefficient.

*DRUJ instability was measured by the ballottement test.

^†^*P* < 0.05.

## Discussion

The arthroscopic repair techniques for TFCC peripheral tears have been revised to make them more straightforward and ligament-specific^2,3,9,21^. This study describes the surgical technique of arthroscopic en Masse repair based on a single-bone-tunnel, two ligament-specific strands, and anchorless repair. This method could simultaneously repair the superficial and deep limbs with each strand that individually tightens the volar or dorsal RUL. Besides, it required only a single-bone-tunnel without an effort to create two tunnels having different angles in the limited area of the distal ulna. After arthroscopic en Masse repair, this study showed improved postoperative clinical outcomes without serious complications.

Although arthroscopic TFCC repair offers the advantages of minimal invasiveness and potentially faster early recovery, it is technically demanding and associated with a substantial learning curve. In contrast, open foveal repair is procedurally more straightforward, and several studies comparing open and arthroscopic TFCC repair have reported similar clinical outcomes and reoperation rates^26,27^. Nevertheless, some studies have reported modest early advantages with arthroscopic repair. Lee et al. observed faster early improvements in postoperative pain and wrist range of motion after arthroscopic foveal repair in their retrospective cohort study with 79 patients (arthroscope = 36, open = 39) with TFCC peripheral tear^28^. In a prospective comparative series of 49 patients, Luchetti et al. found better DASH scores in the arthroscopic-assisted group, whereas other clinical parameters did not differ between groups^27^. Anderson et al. reported a higher incidence of postoperative dorsal ulnar sensory nerve symptoms after open repair (14/39) than after arthroscopic repair (8/36)^26^. Considering these early advantages, the arthroscopic technique should be simplified and standardized to enhance reproducibility and facilitate wider implementation in clinical practice.

Patients with TFCC peripheral tears could injure both superficial and deep limbs together. Previous clinical studies of arthroscopic repair for TFCC peripheral tear patients revealed that TFCC tears involving both superficial and deep limbs (Atzei class 2) occupied 18.8% to 28.6% in intraoperative arthroscopic examination^5,9,29,30^. Together with our finding of 29.2% of Atzei class 2, it is speculated that additional capsular repair for concurrent superficial limb tear is occasionally required during the TFCC foveal repair^31,32^. In these cases, arthroscopic en Masse repair of TFCC, attaching the superficial limb to the capsule using the same strand securing the deep limb to the ulnar fovea, would be beneficial. It could not only simplify the procedure of arthroscopic TFCC repair but also standardize it regardless of combined superficial limb tear.

Recent biomechanical and clinical studies emphasize the ligament-specific repair for dorsal and volar RULs in TFCC peripheral tears. It was first proposed by biomechanical studies advocating that each RUL has its specific physiologic function on the ulnar head translation during forearm rotation^1,33^. Lately, a clinical study by Liu et al. has reported clinical outcomes of arthroscopic ligament-specific TFCC repair in twenty-five patients at a minimum follow-up of 2 years^3^. They supported the necessity of anatomical repair for TFCC peripheral tears with excellent clinical outcomes, although they failed to demonstrate clinical differences between double-limb and single-limb repairs. On the other hand, we believe that two strands during the ligament-specific repair could compress a larger area of TFCC to the footprint than a single-strand repair, and it would be advantageous for the TFCC healing to the ulnar fovea. In the retrospective study of ligament-specific two-strand repair, Liu et al. reported no patient with postoperative DRUJ instability at a minimum of 2-years follow-up, different from other studies with non-ligament-specific one-strand repair showing the re-rupture in 14–18% of patients^30,34^. Our study also showed a negative ballottement test in all patients one year after the surgery; however, a further biomechanical or long-term comparative clinical study should be required to prove the higher healing rate of the ligament-specific repair.

Various arthroscopic techniques to fix the peripheral TFCC to the ulnar fovea have been suggested and modified using different numbers of bone tunnels or different types of suture anchors. The most common method is creating two bone tunnels with K-wire and tied unabsorbable suture over a bone tunnel on the ulna^35^. Instead of making two bone tunnels, Park et al. established one large bone tunnel (over 4 mm) to achieve outside-in repair with different suture angles within the tunnel and had to employ a knotless suture anchor to secure the TFCC at the ulnar aspect of the ulna^8,9^. For the ligament-specific repair demanded two separate strands to suture dorsal and volar RULs, Liu et al. created two bone tunnels again with 4-mm intervals^36^. Regarding a 1.5-mm K-wire drilled to make bone tunnels, creating two bone tunnels within this limited space might be challenging. We figured that two bone tunnels would be unnecessary even in the ligament-specific repair if we could place two strands within one centered bone tunnel, considering the geometry of deep limbs converging to the ulnar fovea. Tying in the middle of a FiberWire during the shuttle relay with a preestablished PDS suture, we could readily set two strands in one bone tunnel and perform the ligament-specific repair only with a single-bone-tunnel.

Some preoperative factors of the patients could affect the poor clinical outcomes after TFCC repair. Previous studies stated some predisposing factors for poorer outcomes, such as increased age, weak grip strength, ulnar positive variance, and higher cross-sectional area of the pronator quadratus, although the descriptive power was weak during the multiple regression analysis (*R*^2^ = 0.217*)*^8,37^. In our study, longer symptom duration (*p* = 0.031) and positive ulnar variance (*p* = 0.045) were associated with lower MWS on multivariable analysis. However, model fit was modest (*R*^2^=0.224) and did not identify robust independent predictors; thus, any observed associations should be regarded as exploratory. A larger population study is required to determine the affecting factors, and it would be helpful to establish a more detailed surgical indication for TFCC repair. Meanwhile, ulnar positive variance has been associated with less favorable outcomes in prior reports and in our cohort; however, there is no consensus on the operative strategy when it coexists with a peripheral triangular fibrocartilage complex tear. In our series, one patient ultimately underwent secondary ulnar shortening osteotomy for persistent ulnar-sided wrist pain after isolated foveal repair. Several studies have also reported that apparent preoperative ulnar positive variance may decrease after foveal repair of the triangular fibrocartilage complex, arguing against routine concomitant ulnar shortening osteotomy in all patients^9^. Accordingly, we perform foveal repair first and reserve ulnar shortening osteotomy as a staged procedure only for patients with persistent ulnar-sided pain.

This study has several limitations. First, it is a retrospective case series without a control or comparative group. Future work should include prospective comparative studies—ideally randomized or matched-cohort designs—and biomechanical investigations to compare stabilizing efficacy among repair techniques and to determine potential advantages of our method, such as improved surgical outcomes, reduced operative time, or lower complication rates. Second, it included a relatively small number of patients and was insufficient to perform a multiple regression analysis. Third, the cohort was relatively young (mean age, 31.1 years) and predominantly involved acute traumatic peripheral TFCC tears, which may limit generalizability to older patients or to degenerative/chronic tears. Fourth, DRUJ stability grading relied on a single-examiner ballottement test. Although the holding technique demonstrates substantial intra- and inter-rater reliability and moderate diagnostic accuracy, sensitivity varies by setting; findings should be interpreted accordingly^38,39^.

## Conclusion

Arthroscopic-assisted en Masse repair for peripheral TFCC tears was associated with improved clinical outcomes at one year or more after surgery, and postoperative ballottement testing revealed no detectable DRUJ laxity. Within this cohort, one-year follow-up precluded assessment of long-term durability; also, applicability to degenerative/chronic peripheral TFCC tears remains undetermined.

## Supplementary Information

Below is the link to the electronic supplementary material.

Supplementary Material 1

Supplementary Material 2

## Data Availability

Data are available on request from Won-Taek Oh (owont@yuhs.ac).

## References

[1] Kleinman, W. B. Stability of the distal Radioulna joint: biomechanics, pathophysiology, physical diagnosis, and restoration of function what we have learned in 25 years. *J. Hand Surg. Am.* **32**, 1086–1106. 10.1016/j.jhsa.2007.06.014 (2007). 17826566 10.1016/j.jhsa.2007.06.014

[2] Nakamura, T., Sato, K., Okazaki, M., Toyama, Y. & Ikegami, H. Repair of foveal detachment of the triangular fibrocartilage complex: open and arthroscopic transosseous techniques. *Hand Clin.* **27**, 281–290. 10.1016/j.hcl.2011.05.002 (2011). 21871351 10.1016/j.hcl.2011.05.002

[3] Liu, B., Arianni, M. & Wu, F. Arthroscopic ligament-specific repair for triangular fibrocartilage complex foveal avulsions: a minimum 2-year follow-up study. *J. Hand Surg. Eur. Vol*. **46**, 270–277. 10.1177/1753193420957901 (2021). 32967517 10.1177/1753193420957901

[4] Srinivasan, R. C., Shrouder-Henry, J. J., Richard, M. J. & Ruch, D. S. Open and arthroscopic triangular fibrocartilage complex (TFCC) repair. *J. Am. Acad. Orthop. Surg.* **29**, 518–525. 10.5435/JAAOS-D-20-00998 (2021). 34078841 10.5435/JAAOS-D-20-00998

[5] Jung, H. S. et al. Arthroscopic transosseous repair of foveal tears of the triangular fibrocartilage complex: A systematic review of clinical outcomes. *Arthroscopy* **37**, 1641–1650. 10.1016/j.arthro.2020.12.209 (2021). 33359818 10.1016/j.arthro.2020.12.209

[6] Omokawa, S. et al. A Biomechanical perspective on distal Radioulnar joint instability. *J. Wrist Surg.* **6**, 88–96. 10.1055/s-0037-1601367 (2017). 28428909 10.1055/s-0037-1601367 PMC5397311

[7] Xiao, A. X. et al. Management of acute distal Radioulnar joint instability following a distal radius fracture: A systematic review and Meta-Analysis. *J. Hand Surg. Glob Online*. **3**, 133–138. 10.1016/j.jhsg.2021.02.005 (2021). 35415552 10.1016/j.jhsg.2021.02.005 PMC8991773

[8] Jung, H. S. et al. Clinical outcomes and factors influencing these outcome measures resulting in success after arthroscopic transosseous triangular fibrocartilage complex foveal repair. *Arthroscopy* **35**, 2322–2330. 10.1016/j.arthro.2019.03.060 (2019). 31351810 10.1016/j.arthro.2019.03.060

[9] Park, J. H., Kim, D. & Park, J. W. Arthroscopic one-tunnel transosseous foveal repair for triangular fibrocartilage complex (TFCC) peripheral tear. *Arch. Orthop. Trauma. Surg.* **138**, 131–138. 10.1007/s00402-017-2835-3 (2018). 29124362 10.1007/s00402-017-2835-3

[10] Matsumoto, T., Tang, P., Fujio, K., Strauch, R. J. & Rosenwasser, M. P. The optimal suture placement and bone tunnels for TFCC repair: A cadaveric study. *J. Wrist Surg.* **7**, 375–381. 10.1055/s-0038-1661361 (2018). 30349749 10.1055/s-0038-1661361 PMC6196092

[11] Park, J. H. & Park, J. W. Arthroscopic transosseous repair for both proximal and distal components of peripheral triangular fibrocartilage complex tear. *Indian J. Orthop.* **52**, 596–601. 10.4103/ortho.IJOrtho_598_16 (2018). 30532299 10.4103/ortho.IJOrtho_598_16 PMC6241056

[12] Mak, M. C. K. & Ho, P. C. Complications after arthroscopic triangular fibrocartilage complex (TFCC) surgery. *J. Hand Surg. Eur. Vol*. **49**, 149–157. 10.1177/17531934231218608 (2024). 38315134 10.1177/17531934231218608

[13] Afifi, A., Abdel-Ati, E. A., Abdel-Wahed, M. & Moharram, A. N. Arthroscopic-Assisted foveal reattachment of triangular fibrocartilage complex tears with distal Radioulnar joint instability: A comparison of suture anchors and transosseous sutures. *J. Hand Surg. Am.* **47**, 507–516. 10.1016/j.jhsa.2022.01.021 (2022). 35341629 10.1016/j.jhsa.2022.01.021

[14] Ma, H. H., Wang, J. P. & Yang, C. Y. Effectiveness of suture anchor and transosseous suture technique in arthroscopic foveal repair of the triangular fibrocartilage complex: a systematic review. *J. Orthop. Surg. Res.* **19**, 72. 10.1186/s13018-024-04530-4 (2024). 38229172 10.1186/s13018-024-04530-4 PMC10790567

[15] Chen, A. C. et al. Results of arthroscopic repair of peripheral triangular fibrocartilage complex tear with exploration of dorsal sensory branch of ulnar nerve. *Open. Orthop. J.* **11**, 525–532. 10.2174/1874325001711010525 (2017). 28694892 10.2174/1874325001711010525 PMC5470059

[16] Zaidenberg, E. E., Roitman, P., Gallucci, G. L., Boretto, J. G. & De Carli, P. Foreign-Body reaction and osteolysis in dorsal lunate dislocation repair with bioabsorbable suture anchor. *Hand (N Y)*. **11**, 368–371. 10.1177/1558944715627632 (2016). 27698643 10.1177/1558944715627632 PMC5030865

[17] Chen, J. S., Paksima, N., Rocks, M. C., Lin, C. C. & Catalano, L. W. Osteolysis Following the Use of Polyetheretherketone Suture Anchors in Hand and Wrist Surgery: A Preliminary Study. *J. Hand Surg. Am.* **50**, 235e231–235e236. 10.1016/j.jhsa.2023.05.024 (2025). 10.1016/j.jhsa.2023.05.024 37542497

[18] Chen, W. J. Arthroscopically assisted transosseous foveal repair of triangular fibrocartilage complex. *Arthrosc. Tech.* **6**, e57–e64. 10.1016/j.eats.2016.09.004 (2017). 28373941 10.1016/j.eats.2016.09.004 PMC5368163

[19] Atzei, A., Rizzo, A., Luchetti, R. & Fairplay, T. Arthroscopic foveal repair of triangular fibrocartilage complex peripheral lesion with distal Radioulnar joint instability. *Tech. Hand Up. Extrem Surg.* **12**, 226–235. 10.1097/BTH.0b013e3181901b1 (2008). 19060683 10.1097/BTH.0b013e3181901b1

[20] Atzei, A. New trends in arthroscopic management of type 1-B TFCC injuries with DRUJ instability. *J. Hand Surg. Eur. Vol*. **34**, 582–591. 10.1177/1753193409100120 (2009). 19620186 10.1177/1753193409100120

[21] Kim, J. P. & Park, M. J. Assessment of distal Radioulnar joint instability after distal radius fracture: comparison of computed tomography and clinical examination results. *J. Hand Surg. Am.* **33**, 1486–1492. 10.1016/j.jhsa.2008.05.017 (2008). 18984328 10.1016/j.jhsa.2008.05.017

[22] Moriya, T. et al. Effect of triangular ligament tears on distal Radioulnar joint instability and evaluation of three clinical tests: a Biomechanical study. *J. Hand Surg. Eur. Vol*. **34**, 219–223. 10.1177/1753193408098482 (2009). 19282400 10.1177/1753193408098482

[23] Nagashima, M. et al. Reliability and validity analysis of the distal Radioulnar joint ballottement test. *J. Hand Surg. Am.*10.1016/j.jhsa.2023.10.006 (2023). 37999702 10.1016/j.jhsa.2023.10.006

[24] Jester, A., Harth, A. & Germann, G. Measuring levels of upper-extremity disability in employed adults using the DASH questionnaire. *J. Hand Surg. Am.* **30**10.1016/j.jhsa.2005.04.009 (2005). 1074 e1071-1074 e1010. 10.1016/j.jhsa.2005.04.009 16182070

[25] Palmer, A. K., Glisson, R. R. & Werner, F. W. Ulnar variance determination. *J. Hand Surg. Am.* **7**, 376–379. 10.1016/s0363-5023(82)80147-0 (1982). 7119397 10.1016/s0363-5023(82)80147-0

[26] Anderson, M. L. et al. Clinical comparison of arthroscopic versus open repair of triangular fibrocartilage complex tears. *J. Hand Surg. Am.* **33**, 675–682. 10.1016/j.jhsa.2008.01.020 (2008). 18590850 10.1016/j.jhsa.2008.01.020

[27] Luchetti, R., Atzei, A., Cozzolino, R., Fairplay, T. & Badur, N. Comparison between open and arthroscopic-assisted foveal triangular fibrocartilage complex repair for post-traumatic distal radio-ulnar joint instability. *J. Hand Surg. Eur. Vol*. **39**, 845–855. 10.1177/1753193413501977 (2014). 23962870 10.1177/1753193413501977

[28] Lee, S. W., Hong, J. J., Sung, S. Y., Park, T. H. & Kim, J. S. Clinical outcomes and failure rate of triangular fibrocartilage complex foveal repair were comparable between arthroscopic and open techniques. *J. Clin. Med.* **13**10.3390/jcm13102766 (2024). 10.3390/jcm13102766 PMC11122638 38792310

[29] Abe, Y., Fujii, K. & Fujisawa, T. Midterm results after open versus arthroscopic transosseous repair for foveal tears of the triangular fibrocartilage complex. *J. Wrist Surg.* **7**, 292–297. 10.1055/s-0038-1641720 (2018). 30174985 10.1055/s-0038-1641720 PMC6117178

[30] Jegal, M., Heo, K. & Kim, J. P. Arthroscopic Trans-osseous suture of peripheral triangular fibrocartilage complex tear. *J. Hand Surg. Asian Pac.* **21**, 300–306. 10.1142/S2424835516400105 (2016). 10.1142/S2424835516400105 27595945

[31] Atzei, A., Luchetti, R. & Braidotti, F. Arthroscopic foveal repair of the triangular fibrocartilage complex. *J. Wrist Surg.* **4**, 22–30. 10.1055/s-0035-1544226 (2015). 25709875 10.1055/s-0035-1544226 PMC4327728

[32] Takagi, T., Nakamura, T. & Fukuoka, M. Arthroscopic capsular repair for triangular fibrocartilage complex tears. *J. Wrist Surg.* **10**, 249–254. 10.1055/s-0040-1721140 (2021). 34109070 10.1055/s-0040-1721140 PMC8169160

[33] Xu, J. & Tang, J. B. In vivo changes in lengths of the ligaments stabilizing the distal Radioulnar joint. *J. Hand Surg. Am.* **34**, 40–45. 10.1016/j.jhsa.2008.08.006 (2009). 19058922 10.1016/j.jhsa.2008.08.006

[34] Shinohara, T. et al. Arthroscopically assisted repair of triangular fibrocartilage complex foveal tears. *J. Hand Surg. Am.* **38**, 271–277. 10.1016/j.jhsa.2012.11.008 (2013). 23351910 10.1016/j.jhsa.2012.11.008

[35] Dunn, J. C., Polmear, M. M. & Nesti, L. J. Surgical repair of acute TFCC injury. *Hand (N Y)*. **15**, 674–678. 10.1177/1558944719828007 (2020). 30762446 10.1177/1558944719828007 PMC7543220

[36] Liu, B. & Arianni, M. Arthroscopic Ligament-specific repair for triangular fibrocartilage complex foveal avulsion: A novel technique. *Tech. Hand Up. Extrem Surg.* **24**, 175–181. 10.1097/BTH.0000000000000292 (2020). 32412983 10.1097/BTH.0000000000000292

[37] Ruch, D. S. & Papadonikolakis, A. Arthroscopically assisted repair of peripheral triangular fibrocartilage complex tears: factors affecting outcome. *Arthroscopy* **21**, 1126–1130. 10.1016/j.arthro.2005.05.024 (2005). 16171639 10.1016/j.arthro.2005.05.024

[38] Nagashima, M. et al. Reliability and validity analysis of the distal Radioulnar joint ballottement test. *J. Hand Surg. Am.* **49**, 15–22. 10.1016/j.jhsa.2023.10.006 (2024). 37999702 10.1016/j.jhsa.2023.10.006

[39] Pickering, G. T., Fine, N. F., Knapper, T. D. & Giddins, G. E. B. The reliability of clinical assessment of distal Radioulnar joint instability. *J. Hand Surg. Eur. Vol*. **47**, 375–378. 10.1177/17531934211054282 (2022). 34727760 10.1177/17531934211054282

