# Supplementary material for: Arthroscopy assisted single bone tunnel two stranded en Masse repair for peripheral triangular fibrocartilage complex tears
Source: Sci Rep. 2025 Dec 29;15:44773. doi: 10.1038/s41598-025-28681-4 (PMC12749120; doi:10.1038/s41598-025-28681-4)
Supplement: Supplementary file 2 — Supplementary Material 2 [file 41598_2025_28681_MOESM2_ESM.docx]

**Supplementary Video 1.** Arthroscopy assisted single bone tunnel two-stranded en Masse repair for peripheral TFCC tear. (1) After traction (5–8 kg) with finger traps, landmarks are marked and standard 3–4 and 6R portals are established. (2) Diagnostic arthroscopy via the 3–4 portal shows an intact central TFCC but a foveal detachment confirmed by trampoline and hook tests. (3) A distal DRUJ portal is created under visualization; a direct foveal portal is then established to expose the deep foveal tear. (4) The foveal bed is freshened with shaver/arthrothermal device to enhance healing. (5) Using a dedicated TFCC guide, a single trans-ulnar bone tunnel is drilled from ~1.5 cm proximal to the ulnar styloid tip to the fovea. (6) A 20-gauge needle delivers a shuttle (2-0 PDS) through the tunnel and across the torn TFCC into the ulnar carpal joint. (7) The shuttle is exchanged for two 2-0 FiberWire sutures; the FiberWire is split into two strands and retrieved through accessory proximal 6R portals. (8) Sutures are tied to achieve en masse foveal repair, tightening both deep and superficial TFCC limbs with the wrist in neutral. (9) Final assessment shows a stable repair (restored trampoline/negative hook tests) and improved DRUJ stability.
